# Exploring the Impact of Inter-Layer Structure on Glass Fiber-Poplar Composite Board: Mechanical and Thermal Properties Analysis

**DOI:** 10.3390/ma18143284

**Published:** 2025-07-11

**Authors:** Jiong Zhang, Shurui Liu, Jinpeng Li, Jixuan Wang, Haoyu Bai, Peng Wei, Tian Liu

**Affiliations:** 1 Key Laboratory of Bio–Based Material Science & Technology, Ministry of Education, Northeast Forestry University, 26 Hexing Road, Harbin 150040, China; 18855053338@163.com (J.Z.); 15124582237@163.com (S.L.); ljp15239943912@163.com (J.L.); hughbhycamp@163.com (H.B.); 2 School of Mathematical Sciences, Ministry of Industry and Information Technology, Beihang University, 9 South Third Street, Beijing 102206, China; wjx3253814211@163.com; 3 Xuzhou Shenghewood Co., Ltd., Banzhuang Industrial Park, Guanhu Town, Pizhou, Xuzhou 221321, China; pzswp@126.com

**Keywords:** glass fiber, poplar veneer, laminated structure, Abaqus finite element simulation, thermal diffusion coefficient

## Abstract

This study presents the design and fabrication of a glass fiber–poplar veneer composite plate, investigating how varying interlayer configurations of glass fiber (single- and double-layer) and the arrangement of poplar veneer layers (odd and even) impact the mechanical and thermal insulation characteristics of these composite plates. Compared to plywood made from natural wood, glass fiber significantly improved the properties of fast-growing poplar plywood. The highest impact strength increased by 3.62 times, while the flexural strength increased by 26.22% and the tensile strength by 29.66%. The thermal diffusion coefficient of the experimental group decreased by 40.74%, indicating better insulation. Interestingly, single-layer glass fiber is superior to a double-layer structure in terms of thermal insulation. An optimal interlayer structure was identified, comprising one veneer layer between two layers of glass fiber cloth, repeated three times. Abaqus 2019 was used for finite element analysis (FEA). The simulation results agree with the experimental data to within 5%. These findings confirm the importance of structural configuration in determining the properties of composite materials, providing a theoretical basis for the structural design of fiber–reinforced composite materials.

## 1. Introduction

In recent years, there has been a gradual shortage of large-diameter wood raw materials. Various types of artificial wood composite panels have been used extensively in the fields of machinery and building materials, such as beams, columns, wall panels, floors, and other building structures. These panels provide stable support and structural protection for buildings. Wood, the most abundant renewable energy source on Earth, has been extensively utilized in architectural and furniture design throughout history [1]. This is due to its distinctive aesthetic qualities, environmental sustainability, and ease of use [2]. However, the anisotropy, porosity, and low density of wood limit its comparison with high-strength materials. Consequently, there is an urgent need to develop sustainable, high-strength, and excellent thermal insulation wood composite materials.

As demonstrated in extant research, the modification of wood composite materials has hitherto been primarily oriented towards enhancing their mechanical and thermal insulation properties. In terms of mechanical properties, researchers like Furuta et al. [3] have explored methods such as double-sided veneer pasting to enhance the mechanical properties of the plate, while Bekhta’s team [4,5] has introduced supplemented nanocellulose to improve the elastic modulus and bending strength of artificial boards. In addition, the mechanical properties of artificial boards can also be enhanced by changing the engraving density [6], improving interface compatibility [7,8,9], and incorporating materials like non-woven fabric [10], glass fiber [11], carbon fiber [12,13,14], and graphite nanosheets (xGnP) [15]. In the realm of thermal insulation performance, studies have highlighted that the impact of adhesive type and dosage [16], the thickness of the plate [17], and the density of plywood [18] are all closely related to the thermal insulation performance of plywood. Chang et al. [19] made cross–layered wood to improve the thermal insulation performance of the plate; Jeon et al. [20] used biochar to enhance the thermal insulation performance of wood; and Chen and his team [21] made multi–functional biomass aerogels with wood waste as raw material to improve the thermal insulation performance. As an example, Zhang’s team [22] has used SiO_2_ aerogel nanoparticles and C_2_H_6_O to prepare artificial boards with good thermal insulation performance.

In recent years, research on laminated composite materials has gradually received attention, and mechanical properties are an important criterion for measuring the quality of laminated composite materials [23,24]. As demonstrated in extant research, laminated composite materials have been extensively utilized in a variety of fields, including aerospace, transportation, construction, and others. The two main matrix phases of composite materials are wood and metal, and the lamination of these with different reinforcement materials can significantly improve the performance of the composite materials [25]. Research on improving the mechanical strength of laminated composite materials shows that laminated composite materials made of linear elastic layers exhibit nonlinear "overall" material properties when subjected to nonlinear deformation [26]. Under nonlinear deformation, the local stress in the layer may not be proportional to the elastic constant of the layer.

Moreover, the lamination of wood veneers with reinforcement materials is a prevalent modification technique. This method is intended to enhance the mechanical properties of wood by eradicating its loose and porous structure and by leveraging the high performance of the reinforcement materials [27]. Glass fiber, a common and effective reinforcement material, has been widely utilized in furniture [7] and various industries. Combining glass fiber with poplar veneer has demonstrated notable improvements in the mechanical strength and thermal insulation of artificial boards due to its simplicity, cost-effectiveness, and accessibility. In the extant literature, the Tungjitpornkull team [28] conducted research on the enhancement of mechanical properties by studying fiber types and fiber orientation angles. Qi, YJ’s team [29] demonstrated the strengthening effect of glass fibers on wood composite materials by conducting calculations on various tree species and internal stresses. In the laminated modification process of wooden boards, the control of the hot pressing process and the selection of adhesive types and amounts are of great significance. Research has demonstrated that the optimal temperature for hot pressing wooden composite boards is 180 °C [30], as this results in the most optimal mechanical properties. However, it should be noted that the polarity of the surface of the wood and glass fiber is in opposition, which can have a detrimental effect on the bonding performance of the two materials, thus leading to the delamination of the board. Epoxy resin adhesive has been demonstrated to effectively overcome the disadvantage of incompatibility and provide good bonding performance. It is evident that current research has fully demonstrated that glass fiber, as a reinforcing material, can significantly enhance the mechanical properties of wood composite materials. Existing research on hot pressing conditions and adhesive dosage control is both extensive and comprehensive. However, there is still a lack of systematic exploration and verification on the influence of composite plate thickness, number of layers, and interlayer structure on material properties. At the same time, existing research focuses on enhancing the mechanical properties of laminated composite materials, and there is a lack of research on thermal properties, which needs further exploration and verification.

The aim of this study is to manufacture composite panels using poplar veneer coated with glass fiber cloth, and to obtain test samples for evaluating mechanical and thermal insulation performance by hot pressing components along the fiber direction. By conducting comparative experiments on wood glass fiber composite materials with different interlayer structures, the influence of interlayer structure ratios on the mechanical and thermal properties of the composite materials can be further explored. Using Abaqus 2019 for finite element experimental simulation will comprehensively analyze the stress distribution of glass fiber composite material sheets under different loads, and ultimately determine the optimal interlayer structure of glass fiber-reinforced veneer composite materials.

## 2. Materials and Methods

### 2.1. Materials

Poplar veneer measuring 150 × 150 × 1.8 mm^3^ was sourced from Yabuli Wood Industry Co., Ltd., Heilongjiang Province, Harbin, China; E–grade fiberglass cloth (primary fibers with an alkali metal oxide content of less than or equal to 0.5% and a single fiber diameter of 20 microns, EWR 200-1000, 150 × 150 × 0.2 mm^3^, Hunan Ba Brothers New Materials Co., Ltd., Jiangsu Province, Suzhou City, China) and ring oxygen resin AB glue (E–44) was obtained from Shanghai Yi Tuo Company, Shanghai, China.

### 2.2. Sample Preparation

**Figure 1:**
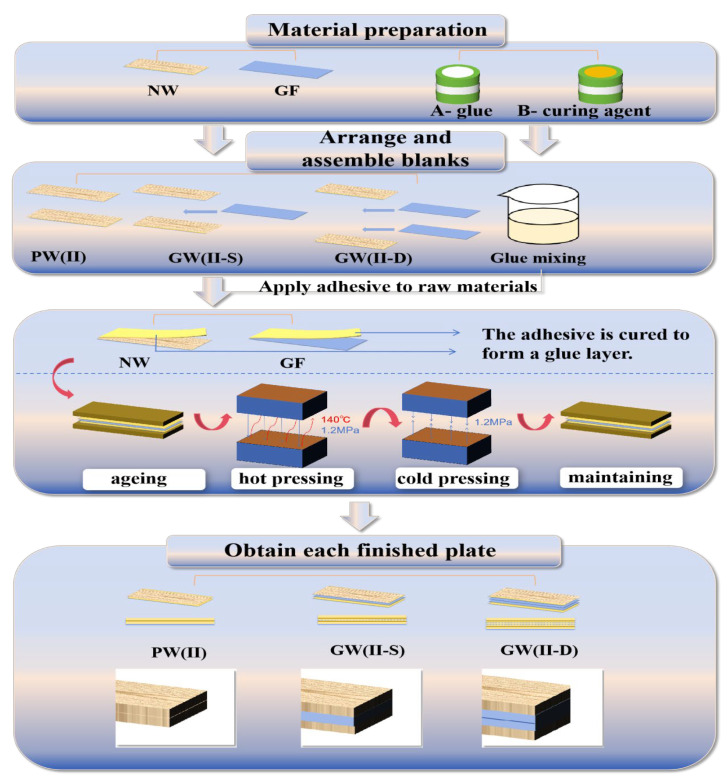
The flow chart for the production and processing of samples.

**Figure 2:**
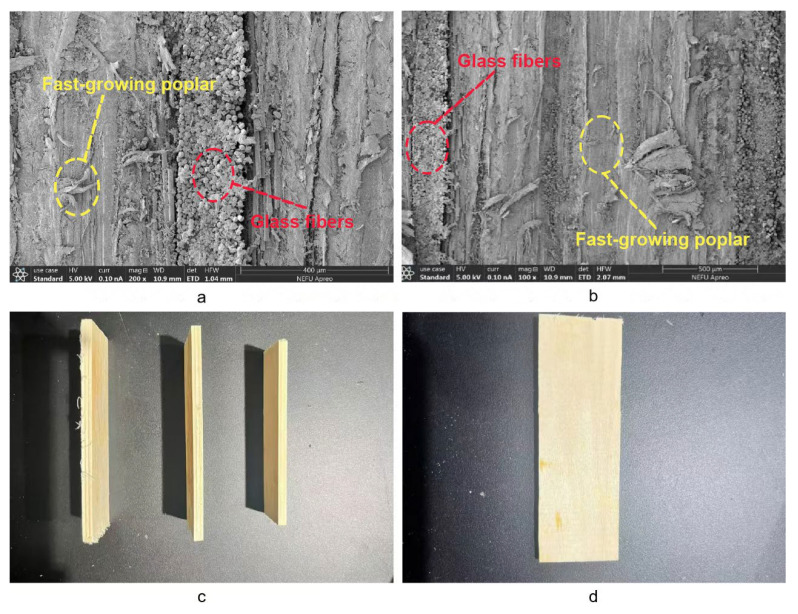
Sample interlayer structure diagram. (**a**,**b**) SEM electron microscope structure diagram. (**c**,**d**) Sample physical diagram.

Figure 1 depicts the flow chart for the production and processing of samples. We carefully selected 45 pieces of air-dried poplar veneer that were devoid of natural defects and exhibited uniform quality. Subsequently, we cut 18 pieces of glass fiber mat to the required dimensions. Then, we arranged the poplar veneer and glass fiber in parallel along the fiber direction and added glass fibers according to a specific interlayer structure to form a multi-layer composite board. The interlayer glass fiber was treated as a single- or double-layer variable, and the number of poplar veneers was set to 2, 3, and 6. In a small beaker, epoxy resin adhesive components A and B were prepared (with a mixing ratio of 0.8:1). The mixture was agitated with a glass rod until it formed a uniformly milky white colloid. We manually applied the adhesive evenly to the contact surface between the veneer (at a rate of 280 g/m^2^) and glass fiber (at a ratio of 220 g/m^2^) using a glue gun, and applied it evenly using a glue brush. Then, we aligned the blanks in the pattern direction and allowed them to rest for a specified period to aid in curing [31]. The composite slab is subjected to a preheating phase in a hot press unit (ZS-406, Dongguan Zhuosheng Machinery Equipment Co., Ltd., Guangdong Province, Dongguan, China) and maintained at a temperature of 140°C and a pressure of 1.2 MPa for a duration of 3 min. Once the target temperature of 140°C was reached, we initiated the formal heating cycle for 20 min. Post–hot pressing, we transferred the slab to a cold press unit (SL-6, Harbin Special Plastic Products Co., Ltd., Heilongjiang Province, Harbin, China) and maintained it at 25 °C for a 5-minute cold pressing phase. Subsequently, we created three sets of blank controls and corresponding single-layer and double-layer experimental groups of glass fibers with varying numbers of veneers. We also produced five test samples in each group that met the size specifications outlined in the testing protocol for various performance tests. These samples were measured in parallel five times, as depicted in Figure 2.

### 2.3. Performance Tests and Characterization

#### 2.3.1. Bending Test

A three-point bending test was conducted on the composite plate sample following the ASTM–D790–03 standard. The bending sample was set to a length of 150 mm and a width of 13 mm, with a span ratio to sample thickness of 16:1. We utilized a universal mechanical testing machine (CMT5504, MTS System Co., Ltd., Shanghai, China) to apply the test at the center of each sample at a speed of 3 mm·min^–1^.

#### 2.3.2. Tensile Test

According to the ASTM–D638–14 standard, the size of each sample was set at a length of 63 mm and a narrow cross-section width of 4.5 mm. At room temperature, the universal mechanical testing machine (CMT5504, MTS System Co., Ltd., Shanghai, China) was used at a test speed of 8 mm·min^–1^ along the wood grain direction where the material was clamped.

#### 2.3.3. Impact Test

The sample underwent an impact test using an impact testing machine (J5, Chengde Jingmi Testing Machine Co., Ltd., Hebei Province, Chengde, China). According to the ISO–179–00 standard, the size of the sample was 75 mm × 7 mm, with a thickness range of 4–10 mm.

#### 2.3.4. Determination of Thermal Conductivity

To determine the overall thermal conductivity of the test sample, we employed the Hot Disk thermal conductivity meter(HOT DISK 2500s, Re’an (Shanghai) Instrument Co., Ltd., Shanghai, China). Subsequently, two specific temperature test points were selected: 25 °C and 40 °C. The test atmosphere during the temperature-up test was nitrogen, with a variable gas flow rate. According to the ASTM E1269–2024 standard [32], the test sample size was 20 mm × 20 mm (length × width), with a thickness greater than 3 mm.

#### 2.3.5. Finite Element Simulation

To enhance the credibility of the experimental data and delve deeper into the internal stress distribution when the composite material undergoes impact, Abaqus 2019 simulation software was used. A three-dimensional solid model was established based on relevant simulation research literature [33], focusing on layered materials, simplified support beams, and stratified bionic beams. The software simulated the mechanical test process of the glass fiber–wood layered composite board within the system [34]. The FEA model divided the analysis object into simple interacting elements (i.e., units) to approach infinite unknown quantities using a limited number of unknown variables.

(1) The Von Mises stress

For structural analysis, the results of the FEA model are generally presented by the Von Mises stress [35]. The Von Mises stress is an equi-effect force based on shear strain energy. Its expression is as follows:

(1) $$\sigma =\sqrt{\frac{{\left(f_{1}-f_{2}\right)}^{2}+{\left(f_{2}-f_{3}\right)}^{2}+{\left(f_{3}-f_{1}\right)}^{2}}{2}}$$

where $f_{1},f_{2},\;and\;f_{3}$ are the first, second, and third main stresses, respectively.

The basis for the analysis of Von Mises stress is the yield condition of Mises, i.e., when the second invariant of partial stress reaches a certain limit, that is, the following:

(2) $$f\left(\sigma_{ij}\right)=J_{2}-k_{2}^{2}=0$$

Here, at the time when the material enters the yield stage, f denotes the yield function, and $k_{2}$ represents the material constant, which can be determined by simple experiments.

(2) Ritz analysis

During the experiment, the glass fiber–poplar composite plate exhibited a moment shape due to the anisotropy of the poplar veneer and fibers [36]. Consequently, the mechanical properties of the resulting composite materials exhibited varying performance across all directions. The stress on different materials of the composite board $\left(\sigma_{1},\sigma_{2},\sigma_{6}\right)$ has a functional relationship with the plane stress related to the geometric direction $\left(\sigma_{xx},\sigma_{yy},\sigma_{xy}\right)$ as depicted in Equation (3):

(3) $$\left(\begin{array}{c} \sigma_{1}\\ \sigma_{2}\\ \sigma_{6} \end{array}\right)=\left(\begin{array}{ccc} {cos}^{2}h & {sin}^{2}h & 2sinhcosh\\ {sin}^{2}h & {cos}^{2}h & -2sinhcosh\\ -sinhcosh & sinhcosh & {cos}^{2}h-{sin}^{2}h \end{array}\right)\left(\begin{array}{c} \sigma_{xx}\\ \sigma_{yy}\\ \sigma_{xy} \end{array}\right)$$

where ***h*** represents the directional angle of the material within the layer.

The bending and compressive properties of the composite board in the fiber direction are considered representative, leading the focus of this experiment to be on studying the bending strength and tensile strength in the grain direction [37].

Ritz’s analysis employs a double series of plane coordinates to shift the line direction, as illustrated in Equation (4):

(4) $$w_{0}\left(x,y\right)=\sum_{m=1}^{M}\sum_{n=1}^{N}A_{mn}X_{m}\left(x\right)Y_{n}\left(y\right)$$

Here, $X_{m}\left(x\right)\;and\;Y_{n}\left(y\right)$ represent the base functions meeting the boundary conditions of the composite board, while $A_{mn}$ is determined by considering the total potential energy in the board. Subsequently, the strain–stress relationship in different directions of the composite board is considered, as outlined in Equation (5)–(7).

(5) $$\varepsilon_{1}=S_{11}\sigma_{1}+S_{12}\sigma_{2}$$

(6) $$\varepsilon_{2}=S_{12}\sigma_{1}+S_{22}\sigma_{2}$$

(7) $$\varepsilon_{6}=S_{66}\sigma_{6}$$

Here, the values of $S_{ij}$ denote all compliance constants, which have the following relationship with the engineering modulus $E_{L},E_{T},G_{LT},\mu_{LT}$:

(8) $$S_{11}=\frac{1}{E_{L}},\;S_{22}=\frac{1}{E_{T}},\;S_{12}=-\frac{\mu_{LT}}{E_{L}},\;S_{66}=\frac{1}{G_{LT}}$$

**Figure 3:**
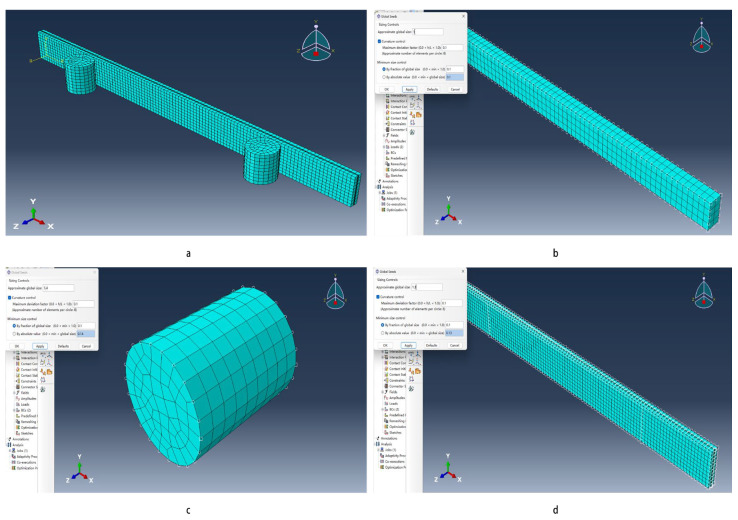
Grid division diagram. (**a**–**c**) Bending sample and pressure roller grid division. (**d**) Stretching model grid division.

(3) Below are the specific operational steps and related parameter settings:
  (A) Model hypothesis:
    To suit the simulation analysis requirements, the following assumptions are made for the composite board model built in Abaqus:
  I. The initial shape of the composite board is assumed to be a cuboid. This simplistic model, though idealized, effectively simulates the stretching experiment by assigning the width to the narrowest part of the spindle shape.
  II. Only the density, Young’s modulus, and Poisson’s ratio are set, with no specific yield stress value. The experimental verification aims to align the yield stress with actual results.
  III. The yielding of the composite board initiates at the geometric center of its lower surface.
  IV. In the bending experiment, the downward displacement of the midline of the upper surface replaces the actual displacement of the cylinder.
  (B) Experimental process and parameter input
  I. Utilizing the Part module and the Create Part function to input length, width, and height for creating the solid model, select the median in each group of experiments with the length, width, and height of the composite board, and then use the Partition Cell function to layer the composite board.
  II. Create three materials in the Property module to represent poplar veneer, glass fiber, and steel, with specific parameters as per Table 1. Establish three isotropic Sections and assign them to the respective Sets.
  III. In the Assembly module, import the sectioned part into the workspace. For the bending experiment, using the Translate Instance function to place the two steel cylinders together with the composite board in a certain position.
  IV. Set up a new analysis step in the Step module, selecting Type as Static, General, enabling the Nlgeom option, setting the initial increment size to 0.1, and keeping the rest as the default.
  V. For stretching experiments, configure contact types using the Interaction module and apply them to the cylinder–composite board intersections.
  VI. Define load and boundary conditions using the Load module based on existing experimental outcomes.
  VII. Employ the Mesh module to the grid in part 3 [38], setting grid sizes to 1 for stretching experiments, 1.3 for the composite plate in bending experiments, and 1.4 for the cylinders, as shown in Figure 3.
  VIII. Create a job in the Job module, submitting it for analysis, and reviewing the results under the Results tab.

**Table 1:** Selection of basic parameters of materials. (Including testing instruments and materials.).

Material	Density (t/mm^3^)	Young’s Modulus (MPa)	Poisson’s Ratio
Poplar veneer	0.65 × 10^−9^	12,000	0.37
Glass fiber	2.5 × 10^−9^	80,000	0.25
Steel	7.8 × 10^−9^	210,000	0.33

## 3. Results and Discussion

### 3.1. Mechanical Properties of Composite Materials

#### 3.1.1. Analysis of Bending Performance

The Duncan model is used to differentiate the experimental data. There exists a significant difference between groups (*p* < 0.05, same below), as summarized in Table 2.

**Figure 4:**
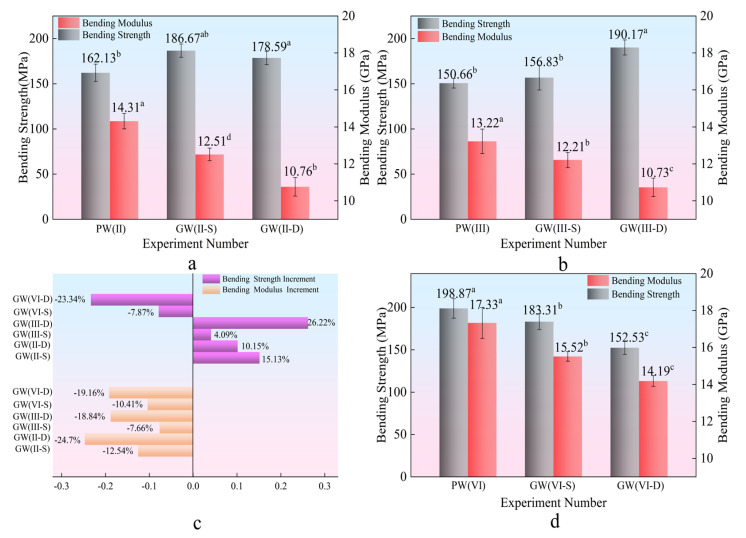
Bending strength and modulus. (**a**,**b**,**d**) Bending strength and modulus Duncan diagram. (**c**) Bending intensity and modulus increment diagram. Note: ^a,b^ and ^c^ represent grade groups with different values from low to high, and the data belonging to different groups have significant differences, representing significant growth or decline. At the same time, two letters, such as ^ab^ represents that their data levels belong to both groups at the same time.

Figure 4 demonstrates the substantial improvement in plate bending strength, enhanced by 26.22%. Notably, the double-layer glass fiber structure outperforms the single-layer configuration in enhancing strength. The bending strength of the composite plate increases with the number of glass fiber cloth layers, indicating a direct correlation between the number of glass fiber layers and the mechanical strength of the composite. However, the bending modulus decreases by 24.70%, suggesting a reduction in the composite plate’s resistance to deformation [39]. This trade–off implies that while glass fibers improve load-bearing capacity, they may compromise the plate’s stiffness.

**Figure 5:**
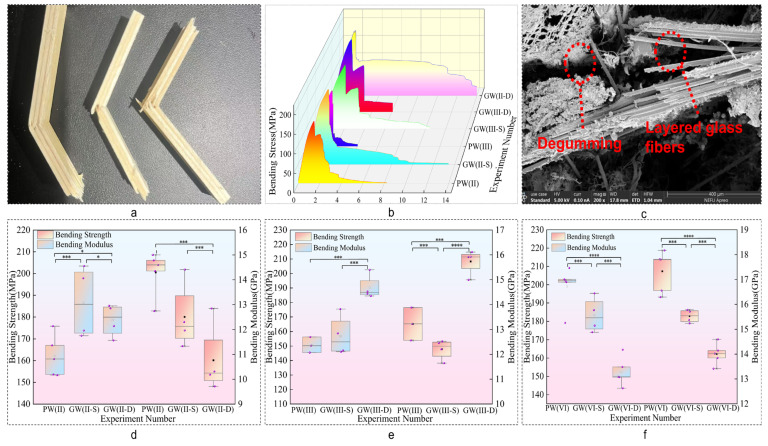
Curved cross-section structure. (**a**) Curved load-bearing deformation diagram. (**b**) Curved stress–strain curve. (**c**) Curved cross-section SEM electron microscope diagram. (**d**–**f**) Curved box diagram. Note: *, *** and **** represent that there are significant differences between them at the statistical level of 10%, 5% and 1%.

According to Figure 5b, the bending load-bearing capacity of the experimental group incorporating glass fibers significantly increases. This enhancement is attributed to the fact that glass fibers may diminish the interlaminar properties of the laminate, leading to a propensity for stratification during the compression process. This stratification disperses stress, prolonging load-bearing duration, but also reduces bending stiffness, making the laminate prone to localized yielding and diminishing the bending modulus [40].

The phenomenon of an increased bending strength and decreased modulus of the test samples is mainly due to the high toughness of glass fiber, which causes a large strain when subjected to bending load. This leads to the delamination fracture of poplar veneer first, while glass fiber still maintains a certain bending strength. The stress–strain curve shows a quadratic upward trend, and the modulus decreases. The interlayer structure of double-layer glass fiber allows for greater strain changes, more significant delamination fracture effects, and a further decrease in the bending modulus of the material.

Figure 5d–f illustrate a 23.34% reduction in bending strength for the PW(VI) group compared to its experimental counterpart, with a notable standard deviation indicating unstable test results. As shown in Figure 5c, the low interfacial compatibility between composite materials contributes to this variability. As the thickness of the plate increases, interfacial adhesion decreases, hindering effective bonding [41]. Excessive plate thickness, under identical pressure, temperature, and time conditions, weakens interlayer bonding, leading to relative slippage and decreased performance. 

#### 3.1.2. Tensile Strength Analysis

**Figure 6:**
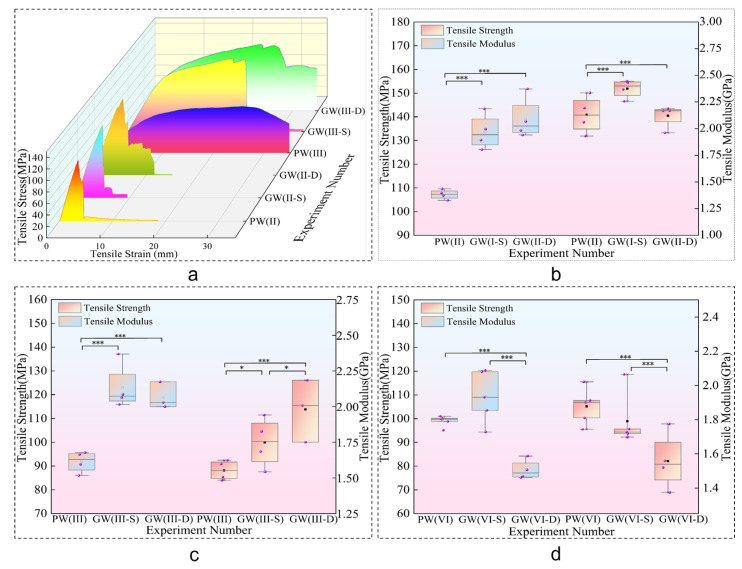
Stretch data chart. (**a**) Stretch stress–strain curve. (**b**–**d**) Stretch box chart. Note: * and *** represent that there are significant differences between them at the statistical level of 10%, 5% and 1%.

In the tensile stress experiment, as shown in Figure 6a, the group incorporating glass fibers exhibited a phased damage response. Initially, there was a weakening of strength followed by an increase in stress, culminating in subsequent damage. This behavior stems from the distinct tensile properties of glass fibers and poplar veneer, coupled with the propensity for debonding between the two materials. The compromised interfacial adhesion allows sliding between layers, resulting in a fiber pull-out phenomena [42]. Consequently, instead of complete destruction, continuous loading induces secondary damage within the composite structure.

The analysis in Figure 6b–d and Table 3 indicates a significant enhancement in the tensile strength (33.94%) and tensile elastic modulus (27.74%) of the composite plate. This enhancement implies that the composite plate, with incorporated glass fibers, can withstand higher maximum stress and exhibits improved stability under tensile stress conditions. Within the interlayer structure, an increase in the number of glass fiber cloth layers results in a relatively consistent rise in tensile strength together with a significant improvement in elastic modulus. This highlights the reinforcing effect of glass fibers on the mechanical properties of the composite material [39].

**Figure 7:**
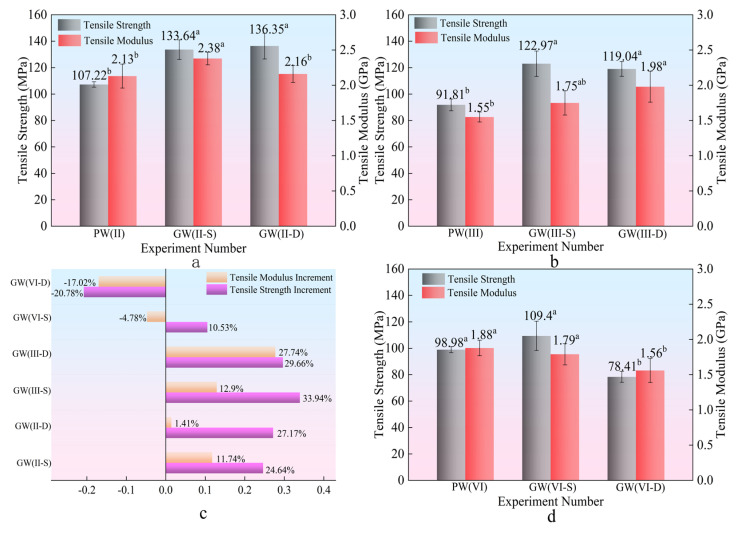
Tensile strength and modulus. (**a**,**b**,**d**) Tensile strength and modulus Duncan diagram. (**c**) Tensile strength and modulus increment diagram. Note: ^a^ and ^b^ represent grade groups with different values from low to high, and the data belonging to different groups have significant differences, representing significant growth or decline.

As depicted in Figure 7, a decremental trend in tensile strength and modulus is observed for the GW(VI) group compared to its experimental counterpart, with a reduction of 20.78%. This decline is attributed to the inherent strength enhancement of the plate with increasing thickness, coupled with a concurrent decline in bonding efficacy—a similar analysis applies to bending. Consequently, the role of glass fiber in augmenting plate performance diminishes with thickness increase, impacting their reinforcing effect. The composite plate’s tensile strength has notably improved by 33.94%, along with a significant enhancement of 27.74% in the tensile elastic modulus, highlighting increased stress resistance and stability under loading conditions when glass fibers are integrated.

Glass fiber has extremely high tensile strength along the fiber direction, which can significantly enhance the tensile properties of laminated composite materials. In addition, due to the uniform stress distribution of glass fibers under tensile stress, the double-layer glass fiber structure has stress evenly distributed on each layer of fibers [13]. Compared with the single-layer structure, its tensile strength and modulus do not have a superposition effect. Therefore, as shown in Figure 7, there is no significant difference in tensile strength and modulus between the two experimental groups.

#### 3.1.3. Impact Strength Analysis

**Figure 8:**
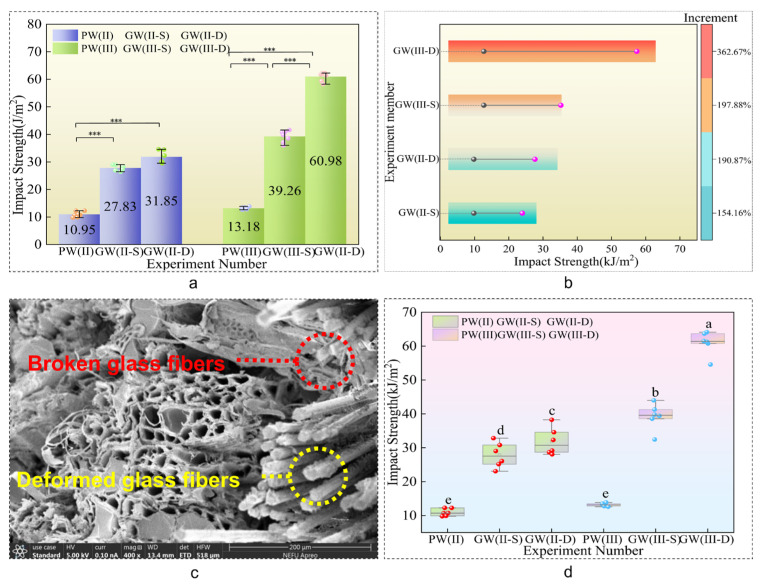
Impact intensity. (**a**) Impact intensity Duncan diagram. (**b**) Incremental diagram. (**c**) Impact section SEM electron microscope diagram. (**d**) Impact box diagram. Note: ^a^, ^b^, ^c^, ^d^ and ^e^ represent grade groups with different values from low to high, and the data belonging to different groups have significant differences, representing significant growth or decline. *** represent that there are significant differences between them at the statistical level of 10%, 5% and 1%.

In the domain of impact testing, the data extracted from Table 4 and Figure 8a,b,d demonstrate substantial increases in impact resistance within the experimental groups compared to their control counterparts. Notably, the PW(II) group exhibited enhancements of 154.16% and 190.87%, respectively, while the PW(III) group experienced even more significant boosts, with increments of 197.88% and 362.67%, respectively. These findings underscore the notable impact of integrating glass fibers on enhancing the impact strength of wooden plates. The addition of glass fibers is a critical factor in significantly bolstering the impact resistance of sheet metal, thereby improving the structural integrity and durability of the composite material under dynamic loading conditions. Furthermore, the overall impact strength escalates markedly with increasing plate thickness and the quantity of incorporated glass fibers. This enhancement in impact resistance is attributed to the synergistic effect of augmented material thickness and glass fiber reinforcement, leading to a more robust resistance to impact forces.

The incorporation of glass fiber into the composite material, as depicted in Figure 8c, induces stratification in the interlayer structure. Upon impact, this stratification leads to localized fiber fractures, significant deformations, and a shift in the plate’s damage mode [43]. This modification allows the material to minimize the affected area and better absorb sudden impact loads. The increased plastic deformation work and fracture work play a role in reducing damage to the laminate, enhancing its ability to withstand external impact loads [40].

### 3.2. Numerical Simulation and Stress Distribution

To assess the accuracy of the experimental data, an error analysis was conducted for each experimental group. As delineated in Table 5, the discrepancies between the experimental data and the finite element simulation results were found to be within a 5% threshold. This alignment between the actual experimental outcomes and the simulation predictions substantiates the authenticity and rationality of the data, affirming the reliability of the experimental procedures and the simulation model employed.

The mean variance loss function was used to calculate the total error of the finite element model, as shown in Equation (9).

(9) $$MSE=\frac{1}{n}\sum_{i=1}^{n}{\left(y_{i}-y_{i}^{’}\right)}^{2}$$

Calculations using Equation (9) revealed mean variance loss function values of 30.035 for bending strength and 13.902 for tensile strength, indicating the model’s strong predictive simulation performance.

**Figure 9:**
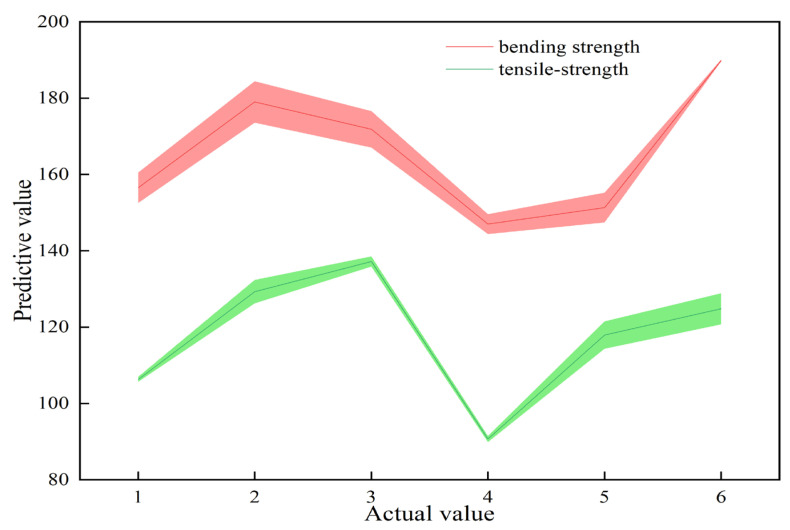
Error diagram.

By using the horizontal coordinates as the actual value, vertical coordinates as the predicted values, and y = x as the reference function, an error band diagram was generated to visually represent the model’s prediction fit and error magnitude, as depicted in Figure 9.

In conclusion, the experimental outcomes align closely with the predictive results, suggesting a high degree of credibility and precision in the predictive methodology. This approach can be deemed a rational and effective method for forecasting the mechanical properties of materials with a minimal loss of accuracy.

**Figure 10:**
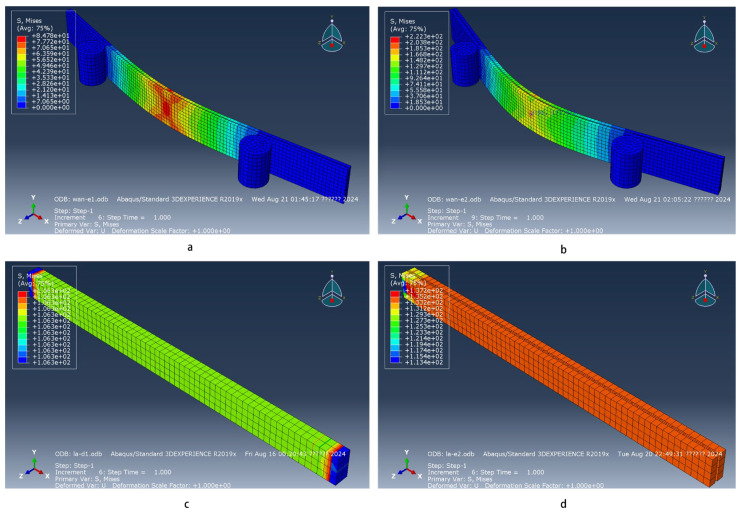
Stress distribution diagram. (**a**,**b**) Bending stress distribution diagram. (**c**,**d**) Tensile stress distribution diagram.

The stress distribution diagram of the test sample under bending and tensile loads is displayed in Figure 10.

In the context of the three-point bending experiment, an analysis of the stress distribution characteristics of the glass fiber–poplar composite plate has been conducted. Specifically, for the lower surface of the composite plate in the fiber direction, a negative correlation between stress magnitude and distance from the neutral axis is observed. Stress increases more rapidly as one moves closer to the neutral axis. An anomalous stress distribution is noted within the small edge region perpendicular to the fiber direction, attributed to localized stress concentrations common in composite materials under bending loads. The stress distribution perpendicular to the fiber direction manifests three distinct patterns, highlighting the anisotropic nature of stress distribution in composites. These findings underscore the complexity of stress distribution in composite materials and the importance of understanding these patterns for the design and analysis of structural components [44]. In the region distal to the neutral axis along the fiber direction, stress magnitude remains essentially constant. Proximal to the neutral axis, stress magnitude exhibits a pronounced variation, adhering to a strong–weak–strong distribution pattern. This pattern is indicative of the stress concentration effects near the neutral axis, where the highest stresses are experienced. Conversely, the stress distribution near the neutral axis follows an inverse weak–strong–weak pattern, signifying a transition from a high-stress concentration to lower-stress regions across the composite material’s cross-section. These observations are crucial for understanding the mechanical behavior of composite materials under bending loads and for the accurate prediction of their structural response [45]. In the same three-point bending experiment context, stress distribution characteristics on the upper surface of the composite plate exhibit a more uniform pattern compared to the lower surface. Along the fiber direction, the magnitude of stress decreases inversely with distance from the neutral axis, intensifying as the distance decreases. Perpendicular to the fiber direction, stress magnitude remains relatively constant. Under conditions of intimate bonding, stress within the glass fiber layer in both experimental groups is generally higher than within the poplar veneer layer, aligning with the stress distribution of the poplar veneer layer [46].

During the tensile testing, stress distribution across the upper and lower surfaces of the poplar veneer layer appears relatively uniform, indicating even load distribution across each segment [47]. The stress distribution pattern within the glass fiber layer mirrors that of the poplar veneer layer. However, non-uniform stress distribution is observed along the fiber direction at the composite board’s ends, arising from the need to clamp the board ends during displacement application—a procedural aspect to consider when evaluating stress distribution patterns.

### 3.3. Analysis of Thermal Insulation Performance

The optimal structures for the main mechanical property tests, PW(II) and PW(III), and their corresponding experimental groups have been analyzed, while the PW(VI) plate with excessive thickness, not suitable for thermal conductivity tests, has been excluded from consideration.

**Figure 11:**
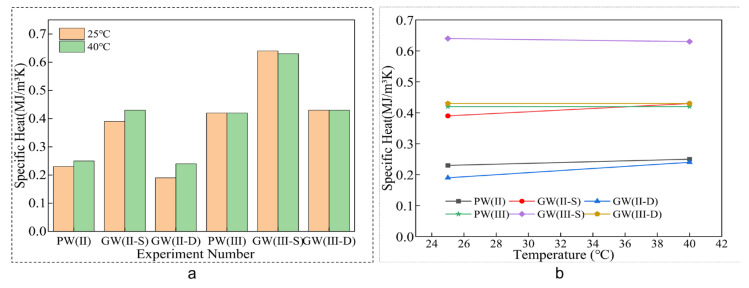
Heat map. (**a**) Column chart. (**b**) Line chart.

As per the data illustrated in Figure 11, the specific heat of the glass fiber composite plate remains constant at both 25 °C and 40 °C. An examination of Table 6 reveals that the single-layer glass fiber cloth configuration exhibits superior thermal properties, with a significant enhancement of 69.57% and 52.38% with specific heat at 25 °C and 40 °C, respectively. In contrast, the double-layer glass fiber cloth experimental group’s thermal properties align closely with those of the control group.

**Figure 12:**
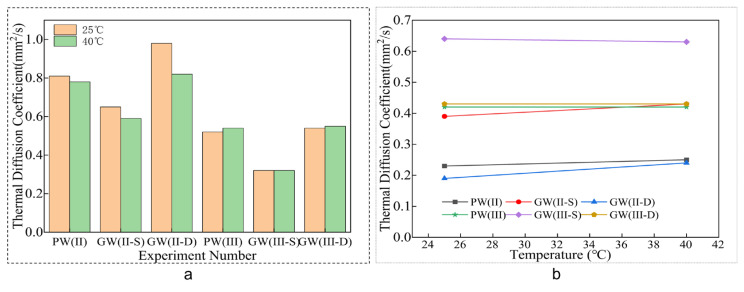
Thermal diffusion coefficient. (**a**) Column chart. (**b**) Line chart.

The data presented in Figure 12 reveal minimal variation in the thermal diffusion coefficient at 25 °C and 40 °C. Comparative analysis with the control group shows a substantial decrease in the thermal diffusion coefficient for the single-layer glass fiber cloth experimental group, with reductions of 19.75% and 38.46% at 25 °C, and 24.36% and 40.74% at 40 °C, respectively, as detailed in Table 7. This trend indicates a progressive decline in the thermal diffusion coefficient with an increase in the number of layers within the composite plate [48].

In summary, the thermophysical properties of the glass fiber composite plate with a single-layer glass fiber cloth configuration exhibit a marked enhancement in specific heat and a significant reduction in thermal diffusion coefficient, as well as an increased heat energy requirement for a 1 °C temperature change and a slower equilibration of the internal plate temperature with external conditions. These findings emphasize the role of the single-layer glass fiber structure in improving the thermal insulation effectiveness of the composite plate [49].

Thermal conductivity, a metric indicating the speed of heat conduction in an object, can be calculated by using Equation (10):

(10) α=λ/ρc

where *λ* denotes the thermal conductivity, *ρ* is the density, and c represents the specific heat capacity. Table 8 shows the thermal conductivity of the composite sheets.

It is evident that the thermal conductivity of glass fiber composite sheets remains essentially constant across both ambient (25 °C) and elevated (40 °C) temperature conditions, with a consistent value of 0.2 W/mK. Notably, the GW(II–S) group exhibited a thermal conductivity of 0.25 W/mK, representing a 31.58% increase compared to the control group. In contrast, the GW(II–D) group’s thermal conductivity aligns with the control group. The PW(III) group and its experimental counterpart demonstrate stable thermal conductivity, fluctuating within a 10% range. These findings underline the influence of composite structure on thermal performance and the variations in thermal conductivity across different configurations of glass fiber composite materials. 

**Figure 13:**
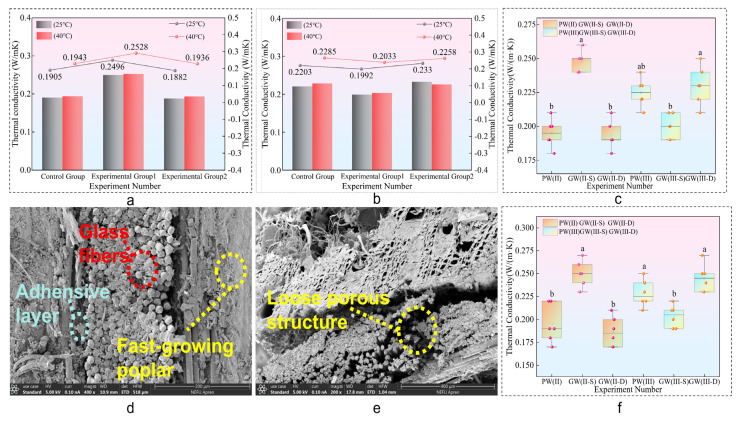
Thermal conductivity. (**a**,**b**) Thermal conductivity column chart. (**d**,**e**) SEM electron microscope diagram. (**c**,**f**) Thermal insulation box diagram. Note: ^a^ and ^b^ represent grade groups with different values from low to high, and the data belonging to different groups have significant differences, representing significant growth or decline. At the same time, two letters, such as ^ab^ represents that their data levels belong to both groups at the same time.

The thermal conductivity of a material directly influences its thermal insulation capabilities. The research findings, as depicted in Figure 13a–c,e, show a consistent thermal conductivity of 0.2 W/mK, with slight increases observed with thicker composite plates due to enhanced interlayer structures and adhesive content. The quantity of epoxy resin adhesive, which possesses a higher thermal conductivity of 1.2 W/mK, is directly proportional to the material’s thermal conductivity. Both glass fiber (0.031 W/mK) and poplar veneer (0.1 W/mK) are low thermal conductivity materials, synergistically reducing the overall thermal conductivity of the composite and enhancing thermal insulation properties. This synergy between glass fiber and poplar veneer underscores the potential for creating high-performance, thermally insulating composite materials applicable in construction and related industries [50].

In the absence of a significant increase in the number of layers of poplar veneer, epoxy resin adhesive, as a highly conductive component, increases its dosage with the increase in the number of layers in the laminated board. The more adhesive layers that heat passes through during interlayer transfer, the more heat is conducted, resulting in an increase in the thermal conductivity of the composite board. At the same time, due to the fact that glass fiber felt is woven from multiple small glass fiber filaments, the porous mesh structure formed by the intertwining of fiber filaments causes a large amount of adhesive to remain trapped in it. The mesh structure of the double-layer glass fiber felt layers stores a large amount of adhesive that has not been stirred evenly, resulting in uneven heating and thermal conduction contact area inside the material, accelerating the thermal conduction of heat in the adhesive layer and improving the thermal conductivity of the composite material.

In this study, the thermophysical properties of the double-layer glass fiber structure, including the thermal conductivity, specific heat, and thermal diffusion coefficient, were found to be essentially invariant compared to the control group. Observations in Figure 13d,e suggest that this stability can be attributed to the loose texture of the glass fiber cloth, facilitating effective bonding with epoxy resin adhesives. The overlapping layers of glass fiber cloth efficiently encapsulate a significant amount of adhesive, leading to an equilibrium state in thermal insulation efficacy and thermal conductivity between the glass fiber cloth and the adhesives.

## 4. Conclusions

In this research, advanced high-strength glass fiber-reinforced composite laminates were fabricated via a hot-pressing molding technique. This process entailed the stratified arrangement of fast-growing poplar veneers and glass fiber cloth in alternating directions, bonded together with epoxy resin adhesive. The resulting composites underwent a comprehensive evaluation of their mechanical and thermal insulation properties. The investigation revealed a trend in mechanical properties where the glass fiber composite plate’s strength initially increased with the number of veneer layers, followed by a subsequent decline. Notably, the GW(III–D) group exhibited optimal performance. The flexural strength peaked at 190.17 MPa, showcasing a notable 26.22% improvement, while the tensile strength reached 139.09 MPa, representing a 29.66% increase. Impact strength saw a significant escalation to 64.57 kJ/m^2^, representing a substantial 362.67% enhancement. The thermal conductivity of the composites remained relatively constant at both 25 °C and 40 °C. With an increase in the number of layers, the thermal diffusivity of the single-layer glass fiber structure decreased by 40.74%, with the specific heat capacity increasing by 69.57%, enhancing thermal insulation efficacy. Statistical analysis using Duncan’s multiple range test revealed significant inter-group differences. Finite element simulations performed with Abaqus indicated a uniform stress distribution in tensile tests and a stress distribution inversely proportional to the distance from the neutral axis in bending tests. In scenarios of adhesive failure, stress predominantly concentrated in the glass fiber layer 43. The closeness between experimental and simulation data within a 5% margin attests to the data’s reliability and the predictive method’s precision, offering a viable approach for forecasting the mechanical properties of the material without experimental loss.

This method effectively mitigates the inherent limitations of low mechanical strength in fast-growing poplar veneers, yielding a secure, sustainable, and high-strength composite material. An optimal interlayer structure was identified, comprising one veneer layer between two layers of glass fiber cloth, repeated three times. This configuration has been demonstrated to engender a substantial enhancement in the mechanical properties of composite materials. The advent of this new interlayer structure composite material signifies its potential for extensive utilization in the domains of construction and furniture manufacturing. The efficacy of this material is predicated on its ability to ensure enhanced structural stability, thereby guaranteeing the quality of the board. It has great application prospects in furniture construction and insulation board manufacturing, and can meet the building needs of a warm winter and cool summer.

In future research, the study of artificial composite panels will receive increasing attention. In the context of reducing pollution and carbon emissions, the ageing resistance and environmental performance of composite panels are of paramount importance. Further exploration is required in order to ascertain effective methods of enhancing the longevity of panels and reducing environmental pollution. This will significantly facilitate the development and application of composite materials.

## Figures and Tables

**Table 2:** Bending strength and modulus.

Group	Average Strength ± Standard Deviation (MPa)	Growth Rate (Comparison) (%)	Modulus Mean ± Standard Deviation (GPa)	Growth Rate (Comparison) (%)
PW(II)	162.13 ± 9.54 ^c^	0.00	14.30 ± 0.90 ^c^	0.00
GW(II–S)	186.67 ± 16.36 ^ab^	15.13	12.50 ± 1.33 ^d^	−12.54
GW(II–D)	178.59 ± 7.43 ^b^	10.15	10.76 ± 1.41 ^e^	−24.70
PW(III)	150.66 ± 5.41 ^c^	0.00	13.22 ± 0.66 ^cd^	0.00
GW(III–S)	156.83 ± 13.68 ^c^	4.09	12.21 ± 0.40 ^d^	−7.66
GW(III–D)	190.17 ± 8.34 ^ab^	26.22	10.73 ± 0.50 ^e^	−18.84
PW(VI)	198.97 ± 11.49 ^a^	0.00	17.33 ± 0.84 ^a^	0.00
GW(VI–S)	183.31 ± 9.45 ^ab^	−7.87	15.52 ± 0.26 ^b^	−10.41
GW(VI–D)	152.53 ± 7.90 ^c^	−23.34	14.01 ± 0.43 ^c^	−19.16

Note: ^a,b^ and other letters indicate the level, and there is a significant difference between the groups corresponding to different levels. The following is the same.

**Table 3:** Tensile strength and modulus.

Group	Average Strength ± Standard Deviation (MPa)	Growth Rate (Comparison) (%)	Modulus Mean ± Standard Deviation (GPa)	Growth Rate (Comparison) (%)
PW(II)	107.22 ± 2.03 ^d^	0.00	2.13 ± 0.17 ^b^	0.00
GW(II–S)	133.64 ± 7.39 ^a^	24.64	2.38 ± 0.09 ^a^	11.74
GW(II–D)	139.09 ± 8.77 ^a^	29.72	2.16 ± 0.11 ^b^	1.41
PW(III)	91.80 ± 4.42 ^e^	0.00	1.55 ± 0.07 ^e^	0.00
GW(III–S)	122.97 ± 9.58 ^b^	33.95	1.75 ± 0.17 ^de^	12.90
GW(III–D)	119.04 ± 5.60 ^bc^	29.67	1.98 ± 0.22 ^bc^	27.74
PW(VI)	98.98 ± 2.30 ^de^	0.00	1.88 ± 0.11 ^cd^	0.00
GW(VI–S)	109.40 ± 11.04^cd^	10.53	1.79 ± 0.15 ^cd^	−4.78
GW(VI–D)	78.41 ± 4.15 ^f^	−20.78	1.56 ± 0.17 ^e^	−17.02

Note: ^a^, ^b^, ^d^, ^e^ and ^f^ represent grade groups with different values from low to high, and the data belonging to different groups have significant differences, representing significant growth or decline. At the same time, two letters, such as ^ab^, ^bc^, ^cd^ and ^de^ represent that their data levels belong to both groups at the same time.

**Table 4:** Impact strength (kJ/m^2^).

Group	Average Value ± Standard Deviation	Lower Limit (95% Confidence)	Upper Limit (95% Confidence)	Growth Rate (Comparison) (%)
PW(II) ^e^	10.95 ± 1.10	9.79	12.11	0.00
GW(II–S) ^d^	27.83 ± 3.69	23.96	31.7	154.16
GW(II–D) ^c^	31.85 ± 4.01	27.64	36.06	190.87
PW(III) ^e^	13.18 ± 0.47	12.68	13.67	0.00
GW(III–S) ^b^	39.26 ± 3.84	35.22	43.29	197.88
GW(III–D) ^a^	60.98 ± 3.42	57.4	64.57	362.67

Note: PW(VI) and its experimental group failed to break under the impact of a 5 J pendulum, which has a very high impact strength. ^a^, ^b^, ^c^, ^d^ and ^e^ represent grade groups with different values from low to high, and the data belonging to different groups have significant differences, representing significant growth or decline.

**Table 5:** Error fitting.

Group	Bending Experimental Data	Bending Simulation Data	Relative Error (%)	Stretching Experimental Data	Stretch Simulation Data	Relative Error (%)
PW(II)	162.13	156.50	3.47	107.22	106.30	0.86
GW(II–S)	186.67	179.00	4.11	133.64	129.30	3.25
GW(II–D)	178.59	171.84	3.78	139.09	137.20	1.36
PW(III)	150.66	146.98	2.44	91.80	90.67	1.23
GW(III–S)	156.83	151.31	3.52	122.97	117.90	4.12
GW(III–D)	190.17	189.84	0.17	119.04	124.80	4.84

**Table 6:** Specific heat (MJ/m^3^K).

Group/Test Temperature Point (°C)	Test Temperature Point (25 °C)	Test Temperature Point (40 °C)
PW(II)	0.23	0.25
GW(II–S)	0.39	0.43
GW(II–D)	0.19	0.24
PW(III)	0.42	0.42
GW(III–S)	0.64	0.63
GW(III–D)	0.43	0.43

**Table 7:** Thermal diffusion coefficient (mm^2^/s).

Group/Test Temperature Point (°C)	Test Temperature Point (25°C)	Test Temperature Point (40°C)
PW(II)	0.81	0.78
GW(II–S)	0.65	0.59
GW(II–D)	0.98	0.82
PW(III)	0.52	0.54
GW(III–S)	0.32	0.32
GW(III–D)	0.54	0.55

**Table 8:** Thermal conductivity (W/mK).

Group/Test Temperature Point (°C)	Test Temperature Point (25 °C)	Test Temperature Point (40 °C)
PW(II)	0.19 ^c^	0.19 ^c^
GW(II–S)	0.25 ^a^	0.25 ^a^
GW(II–D)	0.19 ^c^	0.19 ^c^
PW(III)	0.22 ^b^	0.23 ^ab^
GW(III–S)	0.20 ^bc^	0.20 ^bc^
GW(III–D)	0.23 ^ab^	0.23 ^ab^

Note: ^a^, ^b^ and ^c^ represent grade groups with different values from low to high, and the data belonging to different groups have significant differences, representing significant growth or decline. At the same time, two letters, such as ^ab^ and ^bc^ represent that their data levels belong to both groups at the same time.

## Data Availability

Data is contained within the article.

## Abbreviations

The following abbreviations are used in this manuscript:

PW	plywood
GW	wood with glass fiber
NW	natural wood
GF	glass fiber
xGnP	The number of Graphite Nanosheets/ Polypyrrole Composites

## References

[1] Boonsuk P., Sukolrat A., Bourkaew S., Kaewtatip K., Chantarak S., Kelarakis A., Chaibundit C. (2021). Structure-properties relationships in alkaline treated rice husk reinforced thermoplastic cassava starch biocomposites. Int. J. Biol. Macromol..

[2] Cavdar A.D., Mengeloğlu F., Karakus K. (2015). Effect of boric acid and borax on mechanical, fire and thermal properties of wood flour filled high density polyethylene composites. Measurement.

[3] Furuta N., Hashimoto H., Hirabayashi Y., Hirai T. (2011). Development of Reconstituted Plywood Using Waste Concrete Form Plywood. Mokuzai Gakkaishi.

[4] Bekhta P. (2020). Effect of heat treatment on some physical and mechanical properties of birch plywood. Eur. J. Wood Wood Prod..

[5] Kawalerczyk J., Dziurka D., Mirski R., Siuda J., Babicka M. (2021). Possibility of Use of NCC–Reinforced Melamine–Urea Formaldehyde Adhesive in Plywood Manufacturing. Drv. Ind..

[6] Li C., Li P., Zhu J. (2020). Effect of Curing Pressure on Mechanical Properties of Composite Sandwich Components. China Plast. Ind..

[7] Huang W., Gao X. (2018). Application and Mechanical Properties of Glass Fiber Composites in Furniture. China Plast. Ind..

[8] Jiang H., Kamdem D.P., Bezubic B., Ruede P. (2003). Mechanical properties of poly(vinyl chloride)/wood flour/glass fiber hybrid composites. J. Vinyl Addit. Technol..

[9] Zhang J., Ning L., Yang H., Wu B., Luo S., Rong Z. (2016). Effects of glass fiber content on properties of bamboo flour/ high density polyethylene composites. Acta Mater. Compos. Sin..

[10] Bartoszuk K., Kowaluk G. (2024). Utilization of Fibrous Mat Residues from Upholstered Furniture as Sustainable Fillers in Plywood Production. Materials.

[11] Choi S.W., Li M., Lee W.I., Kim H.S. (2013). Analysis of buckling load of glass fiber/epoxy-reinforced plywood and its temperature dependence. J. Compos. Mater..

[12] Guan M., Liu Y., Zhang Z., Huang Z. (2020). Evaluation of bending performance of carbon fiber-reinforced eucalyptus/poplar composite plywood by digital image correlation and FEA analysis. J. Mater. Sci..

[13] Liu Y., Guan M. (2019). Selected physical, mechanical, and insulation properties of carbon fiber fabric–reinforced composite plywood for carriage floors. Eur. J. Wood Wood Prod..

[14] Liu Y., Guan M., Chen X., Zhang Y., Zhou M. (2019). Flexural properties evaluation of carbon-fiber fabric reinforced poplar/eucalyptus composite plywood formwork. Compos. Struct..

[15] Seo J., Cha J., Kim S., Kim S., Huh W. (2014). Development of the Thermal Performance of Wood-Flooring by Improving the Thermal Conductivity of Plywood. J. Biobased Mater. Bioenergy.

[16] Huang Q., Zheng X., Gao G., He Z., Xie X., Li X. (2019). Studies on the performance and preparation of wood–based permanent formwork with thermal insulation. J. Funct. Mater..

[17] Sun S., Liu L. (2022). Thermal and sound insulation properties of multidimensional–interface extruded tubular particleboards. J. For. Eng..

[18] Bekhta P., Salca E.-A. (2018). Influence of veneer densification on the shear strength and temperature behavior inside the plywood during hot press. Constr. Build. Mater..

[19] Chang S.J., Wi S., Kim S. (2019). Thermal bridging analysis of connections in cross-laminated timber buildings based on ISO 10211. Constr. Build. Mater..

[20] Jeon J., Park J.H., Yuk H., Kim Y.U., Yun B.Y., Wi S., Kim S. (2021). Evaluation of hygrothermal performance of wood-derived biocomposite with biochar in response to climate change. Environ. Res..

[21] Chen H., Deng Q., Hu B., Gao Y. (2022). Flexible curdlan-based aerogels enhanced by wood fibers with ultralow thermal conductivity. Thermochim. Acta.

[22] Zhang Y., Shi J., Ye J., Chen H., Wu Z., Zhan X. (2021). Preparation and performance of hydrophobic thermal insulation plywood for household. J. For. Eng..

[23] Parida S.P., Jena P.C., Dash R.R. (2023). Dynamics of rectangular laminated composite plates with selective layer–wise fillering rested on elastic foundation using higher–order layer–wise theory. J. Vib. Control..

[24] Yuejin H.E., Heng Z. (2008). Experimental Study of Delamination Diagnosis for Composite Laminated Plates. Mater. Rev..

[25] Zhang Z., Zhu P., Zhou S. (2014). Research and Development of Laminated Metal Composite and Electrode Material. Hot Work. Technol..

[26] An H., Singh J., Pasini D. (2017). Structural efficiency metrics for integrated selection of layup, material, and cross-section shape in laminated composite structures. Compos. Struct..

[27] Wang J., Hu Y. (2016). Novel Particleboard Composites Made from Coir Fiber and Waste Banana Stem Fiber. Waste Biomass- Valorization.

[28] Tungjitpornkull S., Sombatsompop N. (2009). Processing technique and fiber orientation angle affecting the mechanical properties of E-glass fiber reinforced wood/PVC composites. J. Mech. Work. Technol..

[29] Qi Y., Xiong W., Liu W., Fang H., Lu W., Batchelor W. (2015). Experimental Study of the Flexural and Compression Performance of an Innovative Pultruded Glass-Fiber-Reinforced Polymer-Wood Composite Profile. PLoS ONE.

[30] Xiao L.G., Ding Y.B., Yan G. (2021). Effect of hot–pressing temperature on characteristics of alkali pretreated reed straw bio–board. J. Wood Chem. Technol..

[31] Lin B., Miao Y., Li R., Jin X., Li M., Liu Z. (2019). Acoustic vibration properties of birch veneer/glass fiber composites. J. Beijing For. Univ..

[32] Lauermannova A.-M., Pavlikova M., Pavlik Z., Pivák A., Jiříčková A., Sklenka J., Záleská M., Růžička K., Jankovský O. (2022). Magnesium oxychloride cement with phase change material: Novel environmentally-friendly composites for heat storage. J. Mater. Res. Technol.-JmrT.

[33] Kim H.-J., Yoon K., Lee P.-S. (2020). Continuum mechanics based beam elements for linear and nonlinear analyses of multi-layered composite beams with interlayer slips. Compos. Struct..

[34] Hundley J.M., Yang J.-M., Hahn H.T., Facciano A.B. (2008). Bearing Strength Analysis of Hybrid Titanium Composite Laminates. AIAA J..

[35] Zhang C., Huang J., Li X., Zhang C. (2020). Numerical Study of the Damage Behavior of Carbon Fiber/Glass Fiber Hybrid Composite Laminates under Low–velocity Impact. Fibers Polym..

[36] El Mahi A., Assarar M., Sefrani Y., Berthelot J.-M. (2008). Damping analysis of orthotropic composite materials and laminates. Compos. Part B: Eng..

[37] Zheng H., Huang Z., Tang S. (2004). Strength Prediction of Composite Laminates by Finite Element Analysis Based on Nonlinear Constitutive Relation. Fiber Reinf. Plast. Compos..

[38] Teng X., Zhang Y. (2014). Nonlinear finite element analyses of FRP-strengthened reinforced concrete slabs using a new layered composite plate element. Compos. Struct..

[39] Zhao J., Xu Z., Wang J., Zhang S. (2014). Influence of fiber–glass on mechanical properties of composite laminates. J. Beijing For. Univ..

[40] Lei Z., Ma J., Sun W., Yin B., Liew K. (2023). Low-velocity impact and compression-after-impact behaviors of twill woven carbon fiber/glass fiber hybrid composite laminates with flame retardant epoxy resin. Compos. Struct..

[41] Cheng F., Hu Y., Li L. (2015). Interfacial properties of glass fiber/unsaturated polyester resin/poplar wood composites prepared with the prepreg/press process. Fibers Polym..

[42] Várdai R., Lummerstorfer T., Pretschuh C., Jerabek M., Gahleitner M., Faludi G., Móczó J., Pukánszky B. (2020). Comparative study of fiber reinforced PP composites: Effect of fiber type, coupling and failure mechanisms. Compos. Part A Appl. Sci. Manuf..

[43] Xiao J., Shi H., Tao L., Qi L., Min W., Zhang H., Yu M., Sun Z. (2020). Effect of Fibres on the Failure Mechanism of Composite Tubes under Low-Velocity Impact. Materials.

[44] Pan Q., Cui H., Cai X., Zhuang J., Yang Z. (2017). Experimental Research and Parameter Analysis of Flexural Behavior of Aluminum Alloy Formwork. Ind. Constr..

[45] Wei X., Qiu C., Liu L., Liang H., Pan L., Liang Q. (2014). Dynamic Mechanical Properties of Long Glass Fiber Reinforced ABS Composite. Plastics.

[46] Glouia Y., Chaabouni Y., El Oudiani A., Maatoug I., Msahli S. (2019). Finite element analysis of mechanical response of cellulosic fiber-reinforced composites. Int. J. Adv. Manuf. Technol..

[47] Xu L., Zhao J., Zhang X., Shi M., Wang Z. (2022). Finite Element Analysis of Flexural Behavior of Shape Memory Alloy Hybrid Composites Laminates. Polymers.

[48] Sun J., Cai F., Tao D., Ni Q., Fu Y. (2021). Enhanced Thermal Insulation of the Hollow Glass Microsphere/Glass Fiber Fabric Textile Composite Material. Polymers.

[49] Dong X., Zhang Q., Lan Y., Zeng Q., Fan M., Chen L., Zhao W. (2022). Preparation and characterization of vacuum insulation panels with hybrid composite core materials of bamboo and glass fiber. Ind. Crop. Prod..

[50] Zhao W., Yan W., Zhang Z., Gao H., Zeng Q., Du G., Fan M. (2022). Development and performance evaluation of wood-pulp/glass fibre hybrid composites as core materials for vacuum insulation panels. J. Clean. Prod..

